# The Effect of 6-Week Combined Balance and Plyometric Training on Change of Direction Performance of Elite Badminton Players

**DOI:** 10.3389/fpsyg.2021.684964

**Published:** 2021-06-10

**Authors:** Zhenxiang Guo, Yan Huang, Zhihui Zhou, Bo Leng, Wangcheng Gong, Yixiong Cui, Dapeng Bao

**Affiliations:** ^1^Sports Coaching College, Beijing Sport University, Beijing, China; ^2^Department of Physical Education, Nanjing University of Aeronautics and Astronautics, Nanjing, China; ^3^Beijing Research Institute of Sports Science, Beijing, China; ^4^China Institute of Sport and Health Science, Beijing Sport University, Beijing, China; ^5^AI Sports Engineering Lab, School of Sports Engineering, Beijing Sport University, Beijing, China

**Keywords:** balance training, plyometrics, change of direction, badminton athletes, badminton

## Abstract

The study aimed to investigate the effect of combined balance and plyometric training on the change of direction (COD) performance of badminton athletes. Sixteen elite male badminton players volunteered to participate and were randomly assigned to a balance-plyometric group (BP: *n* = 8) and plyometric group (PL: *n* = 8). The BP group performed balance combined with plyometric training three times a week over 6 weeks; while the PL group undertook only plyometric training three times a week during the same period. Meanwhile, both groups were given the same technical training. All participants were tested to assess the COD ability before and after the training period: Southeast Missouri (SEMO) test and 5-0-5 test, dynamic balance ability (Y-Balance test, YBT), and reactive strength index (RSI). Repeated-measure ANOVA revealed that after the intervention there was a significant time × group interaction for 5-0-5 COD test, YBT of both legs and RSI (*p* < 0.05, partial η^2^ = 0.26–0.58) due to the better performance observed at post-test compared with a pre-test for the BP group [effect size (ES) = 1.20–1.76], and the improvement was higher than that of the PL group. The change in SEMO test did not differ between BP and PL (*p* < 0.159, partial η^2^= 0.137), but the magnitude of the with-group improvement for BP (ES = 1.55) was higher than that of PL (ES = 0.81). These findings suggest that combined training could further improve the COD performance of badminton athletes than plyometric training alone and might provide fitness trainers a more efficient COD training alternative.

## Introduction

Badminton is one of the fastest racket sports in the world and is highly competitive and dynamic (Phomsoupha and Laffaye, 2015). During the match, the players perform 6–12 strokes within a rally duration ranging from 6 to 10 s. Due to the fast speed of the shuttlecock and high hitting frequency, the sport exerts a great demand on the abilities of player to run, accelerate, decelerate, jump, lunge, and change direction (Laffaye et al., 2015; Lee and Loh, 2019). A previous study showed that among all physical capacities, the COD performance served as the best physical predictor of badminton excellence (r = 0.74) (Hughes and Cosgrove, 2006) so that badminton players maximize such ability to enhance on-court success. Until now, several tests for COD ability have been widely used for badminton assessment, such as the Hexagon test, the 5-0-5 COD test, and the Modified SEMO test, and they were verified as representative methods to determine on-court performance of players (Jeyaraman et al., 2012; Ozmen and Aydogmus, 2016; Wong et al., 2019).

In terms of training programs, traditional resistance training has been used to improve the power and COD ability (Brughelli et al., 2008). However, recent studies have revealed that plyometric training and combined plyometric and resistance training presented greater efficiency in improving these abilities (Asadi et al., 2016; Fischetti et al., 2018, 2019). The actual mechanism of plyometric exercise is a lengthening (eccentric contraction) of the muscle-tendon unit followed directly by a shortening or concentric contraction, otherwise termed as a stretch-shortening cycle (SSC) (Markovic and Mikulic, 2010). In practice, it consists of exercises related to jumping, hopping, and skipping with multi-joint actions on stable or unstable surfaces (Negra et al., 2017a,b). While numerous studies have proved that plyometric training could be applied to improve the COD ability in players in racket sports like tennis (Salonikidis and Zafeiridis, 2008; Barber-Westin et al., 2010; Fernandez-Fernandez et al., 2016, 2018), few attempts have been made for badminton (Lim Joe et al., 2012; Majeed and Latheef, 2016; Middleton et al., 2016). Nonnato and colleagues have recently found that plyometric training cannot improve the 5-0-5 COD test performance of professional female soccer players due to a merely small effect on the COD with 180° angle (Nonnato et al., 2020), and such movement is frequently involved in a badminton match. Therefore, the effect of plyometric training on the COD ability of badminton players remains to be unveiled.

The previous finding has evidenced the importance of balance for the COD performance in that it helps players control the center of gravity (COG) during the accelerations and decelerations phase (Rouissi et al., 2018). For badminton players, balance also plays an important role in addressing issues of controlled COG and other situations challenging their balance, such as twisting movements (particularly of the pivot foot) during jump smash and offensive and defensive attacks. It is logical to undertake balance training to improve movement performance in badminton, particularly COD performance. Previous studies have explored the effect of combined plyometric and balance training on professional female basketball athletes and young soccer players (Makhlouf et al., 2018; Muehlbauer et al., 2019; Bouteraa et al., 2020). The results showed that such a combined program could produce greater performance improvements in balance, power, and COD, as opposed to the single plyometric intervention. However, it was suggested that immaturity or a lack of optimal balance capabilities might compromise the plyometric training adaptations (Bouteraa et al., 2020).

Despite a growing body of literature on combined training in other sport modalities and athletes, there is a paucity of research related to the applications of such training method in badminton. Therefore, it is unclear whether combined training could lead to greater improvement of COD ability in badminton players, or to mention the generalizability of results and practical recommendations from other studies. In order to explore refined fitness programing, the current study was aimed to investigate the effect of 6-week combined balance training and plyometric training on COD performance in elite badminton athletes. We hypothesized that the combined training protocol would further increase the COD and balance performance in badminton athletes when compared with plyometric training alone.

## Materials and Methods

### Participants

**Figure 1:**
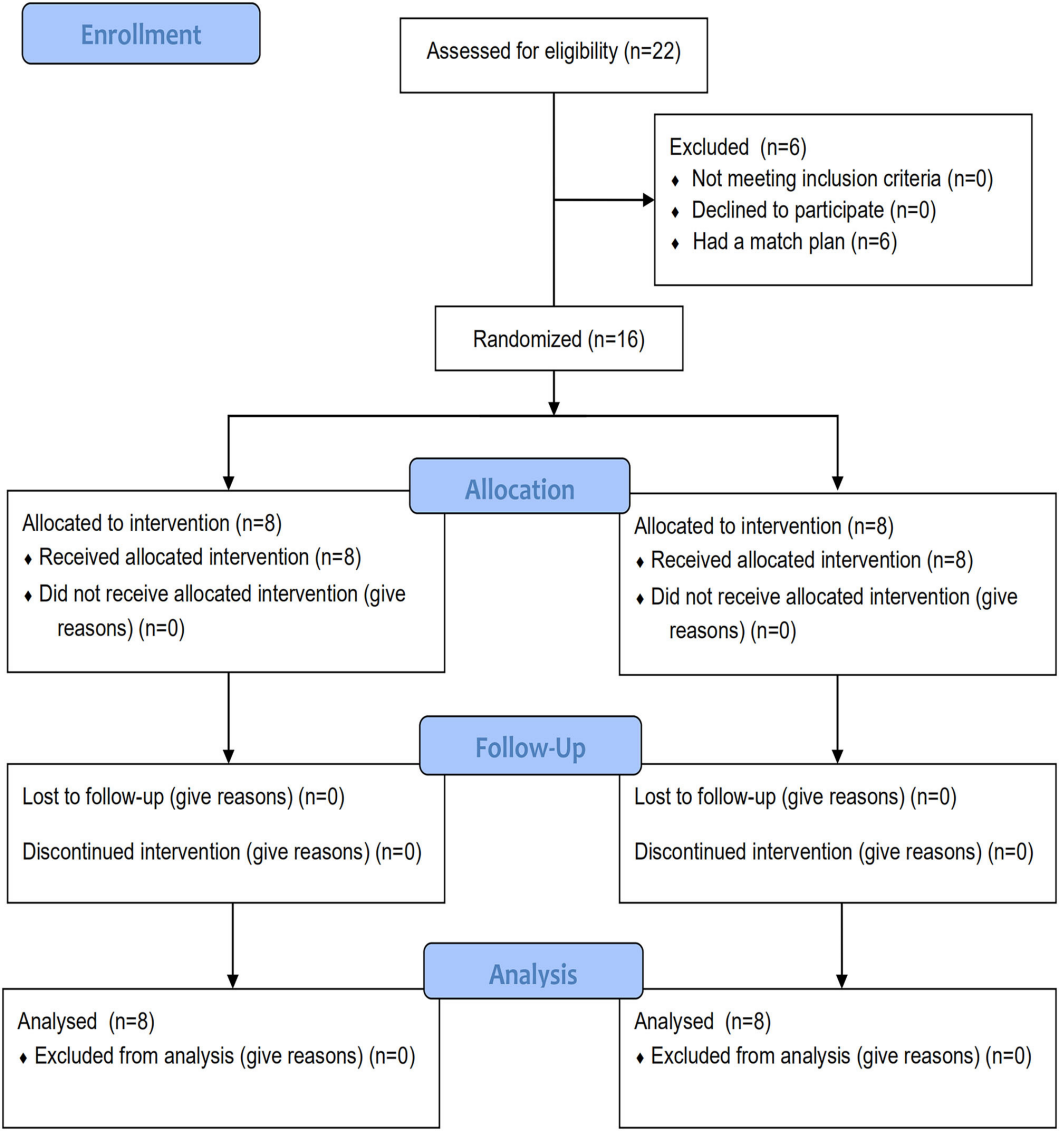
Flow diagram of the participants recruitment process.

Sixteen male elite badminton players (eight players had played the quarterfinalists of national youth games and the rest had played the finals at the provincial level) were recruited in this study. The participants belonged to the same club and were physically healthy, free from severe lower-body injuries related to anterior cruciate ligament (ACL), hamstring, meniscus, and ankle, or any medical and orthopedic problems. All players were right-handed and undertook three training sessions per week with each session formed by 2–3 h of technical and physical training drills (See Figure 1 for participante recruitment process). All participating players volunteered for random allocation to either balance and plyometric training group (BP, *n* = 8) (age: 20.5 ± 1.1 years, height: 177.8 ± 5.1 cm, weight: 68.1 ± 7.2 kg, and training experience: 11.4 ± 1.4 years) or a control group (PL) (age: 19.1 ± 2.2 years, height: 179.1 ± 6.1 cm, weight: 69.88 ± 8.94 kg, and training experience: 10.6 ± 1.1 years) that performed only plyometric training regimen (*n* = 8). There were no statistically significant differences between the groups in these personal characteristics. Before data collection, the participants were informed about the benefits and possible risks associated with the study, and the participants provided written informed consent to participate. The players were in their normal routine of diet and had caffeine-free beverages during the whole study period. The study protocol was approved by the Beijing Sport University Institutional Research Commission (Approval number: 2020008H), and all procedures were conducted in accordance with the Declaration of Helsinki.

### Procedures

All experimental training programs were conducted along with a weekly technical training routine. Participants from the BP and PL groups followed a balance combined with a plyometric training program (40 min of plyometrics and 20 min of balance training) three times per week with 24–48 h of recovery between each training session. In order to control the 20-min balance training protocol, the PL group was required to perform the same drills as the BP group. However, unlike the latter that undertook all the exercises under unstable conditions (i.e., BOSU ball, Swiss ball, and Balance pad), they practiced on the floor. Before the commencement of the study and the initiation of testing, all players completed a 2-week trial period (three sessions/week) in order to become familiarized with the physical training programs during the formal experimental course of the study. A detailed description of balance and plyometric training protocols and biweekly progression are presented in Tables 1, 2. During each session, players received consistent instructions from certified strength and conditioning coaches on proper techniques for agility drills, balance exercises, plyometric exercises, and landing. All the protocols were designed and supervised by one of the authors, who is an experienced researcher in strength and conditioning, and a fitness trainer with a master degree in strength and conditioning, who works as the fitness coach for the Chinese National Junior Team (U-17) of Badminton and collegiate team of badminton and has been certified by the State General Administration of Sport.

**Table 1:** The balance training program for balance-plyometric (BP) (combined training) group.

**Exercises**	**The first stage (1–2 weeks)**	**The second stage (3–4 weeks)**	**The third stage (5–6 weeks)**
Stand on the balance board exercise	Static standing on the board with two legs (3 sets: 30 s/set)	Static standing on the board with two legs and eyes closed (3 sets: 30 s/set)	Squat on the plate with eyes closed (3 sets: 10 reps/set)
Supine straight leg bridge on Swiss Ball	Isometric supine straight leg bridge on Swiss Ball (3 sets: 30 s/set)	Isometric supine single-leg bending bridge on Swiss Ball (3 sets: 30 s/set)	Dynamic supine single-leg bending bridge on Swiss (3 sets: 10 reps/set)
Side-plank with inflated balance disc	Side-plank with inflated balance disc with elbow (3 sets: 30 s/set)	Side-plank with inflated balance disc and the non-supporting leg stretches backward (3 sets: 10 reps/set)	Side-plank with inflated balance disc and the non-supporting leg stretches backward with elastic band (3 sets: 10 reps/set)
Lunge squat on BOSU ball	Lunge squat on BOSU ball (3 sets: 10 reps/leg/set)	Lunge squat on BOSU ball and inflated balance disc (3 sets: 10 reps/leg/set)	Lunge squat on BOSU ball and inflated balance disc with 5 kg dumbbells (3 sets: 10 reps/leg/set)
Airex® Balance-pad Elite exercise	Single-leg squat with balance-pad (3 sets: 10 reps/leg/set)	Single-leg standing with balance-pad and the non-supporting leg stretches backward (3 sets: 12 reps/leg/sets)	Single-leg support with balance-pad elite and the non-supporting leg stretches backward with elastic band (3 sets: 12 reps/leg/sets)
Rest	Between exercise: 60 s Between sets: 3 min		

**Table 2:** The plyometric training program for BP and plyometric (PL) training group.

**Exercises**	**The first stage (1–2 weeks)**	**The second stage (3–4 weeks)**	**The third stage (5–6 weeks)**
Front barrier jump (6 hurdles)	Double-leg front barrier jump (15 cm) (3 sets: 10 reps/set)	Single-leg front barrier jump (15 cm) (3 sets: 5 reps/leg/set)	Single-leg front barrier jump (30 cm) (4 sets: 5 reps/leg/set)
Lateral high-knees with hurdles	4-hurdle (15 cm) (3 sets: 2 reps/set)	6-hurdle (30 cm) (3 sets: 4 reps/set)	6-hurdle (30 cm) (3 sets: 6 reps/set)
Lateral barrier jump	Double-leg jump (15 cm) (3 sets: 10 reps/set)	Double-leg jump (30 cm) (3 sets: 12 reps/set)	Single-leg jump (30 cm) (3 sets: 15 reps/leg/set)
Depth jump	Jump with 20 cm box (3 sets: 8 reps/set)	Jump with 30 cm box (3 sets: 8 reps/set)	Jump with 40 cm box (3 sets: 8 reps/set)
Multi-direction jumps with hurdles	Triangle jump with double-leg (3 hurdles) (3 sets: 6*3 reps/set)	Square jump with single-leg (4 hurdles) (3 sets: 8*3 reps/set)	Hexagon jump with single-leg (6 hurdles) (3 sets: 12*3 reps/set)
Intensity and number of contact with ground	Low intensity 144	Middle intensity 234	High intensity 325
Rest	Between exercise: 60 s Between sets: 3 min		

### Test Program

Data of COD testing were collected before and after the implementation of the 6-week training intervention at the indoor sports science center and badminton court of local institution of authors. The testing consisted of Modified SEMO Test, Modified 5-0-5 COD test, Y-balance test (YBT), and reactive strength index (RSI) test, and they were performed and completed within 1 day. Players were already accustomed to the testing procedures used in this research as they routinely performed these tests in the club. Prior to testing, participants completed a warm-up that included a 5-min dynamic stretching, 8-min movement integration, and 2-min neural activation. Post-training testing was performed 3 days after the last training session to ensure optimal recovery, and no intensive training was implemented 24 h before testing to prevent the effects of fatigue. Each testing was completed at the same time of the day, at the same site, with the same sporting shoes, and surveilled by the same investigators. The set of tests and measurements were detailed in the following context:

### Modified SEMO Test

**Figure 2:**
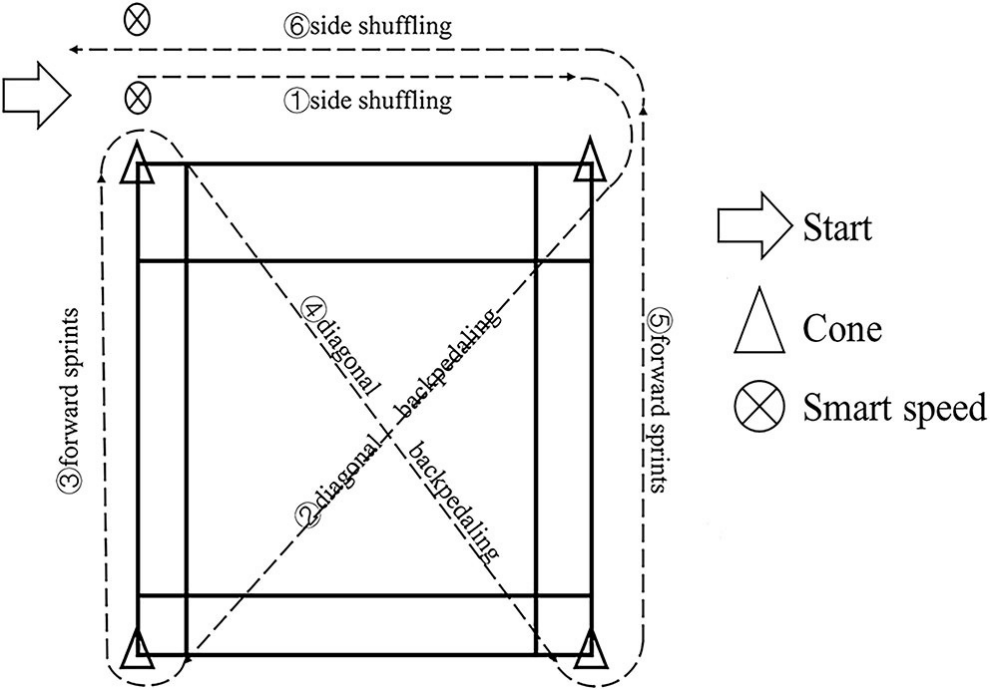
Modified Southeast Missouri (SEMO) test.

This test evaluates the capacity of making quick changes of direction, forward sprints, diagonal backpedaling, and side shuffling, which are the most frequent movements performed during badminton matches (Jeyaraman et al., 2012). The test area was set up on the half of the badminton court with a length of 6.70 m and a width of 6.10 m. Four cones were placed on four corners of the court to mark each site of change of direction (see Figure 2). At each trial, participants first started side shuffling (facing the court) on instructions of the coach, and then back-pedal diagonally across the court, sprint forward, back-pedal diagonally across court again, sprint forward, and finally side-shuffle (facing the court) to the finishing point. Test times were recorded using SmartSpeed PT Timing Gate System (Fusion Sport, Coopers Plains, Australia), which was installed at the start/finish point of the test. Three consecutive trials were performed and separated by 2 min of passive rest, with the highest values being recorded for analysis (intraclass correlation coefficients of a two-way random model: 0.85, 95% CI: [0.74, 0.92]).

### Modified 5-0-5 COD Test

**Figure 3:**
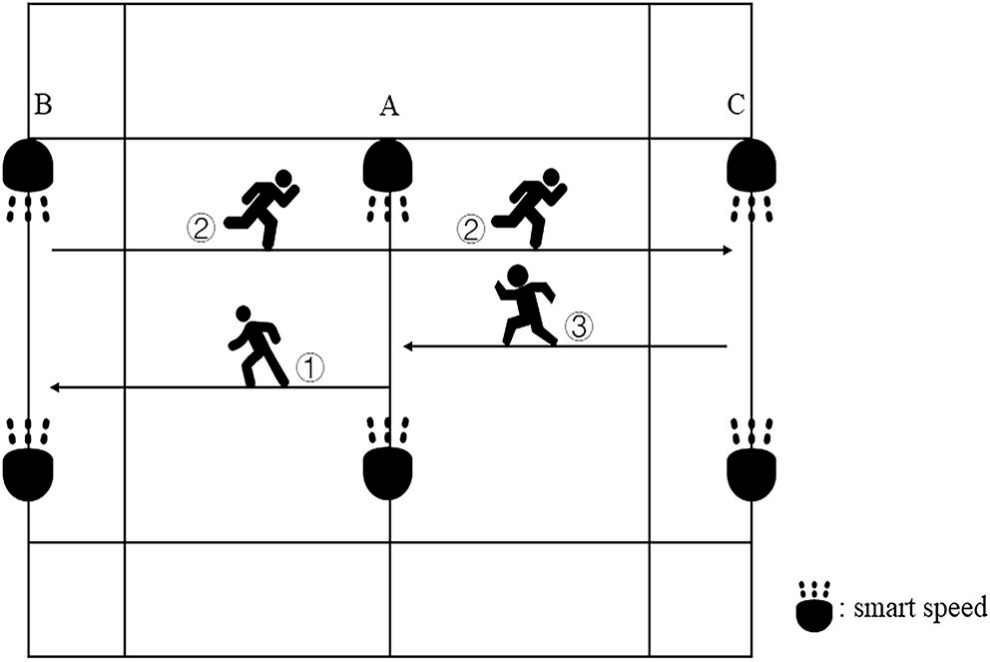
Modified 5-0-5 COD test.

The 5-0-5 COD test is widely used to evaluate the COD ability of athletes in various sports, which is very suitable for those sports that require short-distance acceleration and COD, such as basketball, rugby, tennis, and badminton (Hughes and Bopf, 2005). Due to the short moving distance in the badminton court, players often involve in COD with 180° angle, which is highly similar to the 5-0-5 COD test. In order to suit badminton specifically, the researcher adjusted the running distance of the test to be similar to the distance inside the badminton court, as shown in Figure 3. Two cone buckets A were placed symmetrically at the starting line, then two other cone buckets B were placed at a position 3 m to the right of the parallel A point, and finally, the cone bucket C was placed at a position 3 m to the left of the parallel A point. Smart Speed (Fusion Sport, Coopers Plains, Australia) was placed behind each pair of cones. After hearing the command “Ready, start,” the participants made a quick right turn and ran from A to B. Upon arrival, the participant quickly turned around and sprinted to C, then quickly turned around and ran back to A. Each participant performed three tests, and the best of the three tests was the final valid score. There was a 5–10 min recovery period between each test. Smart Speed was automatically timed at the beginning and the end of the test. Three consecutive trials were performed and separated by 2 min of passive rest, with the highest values being recorded for analysis (intraclass correlation coefficients: 0.88 [0.79, 0.94]).

### Y-Balance Test

**Figure 4:**
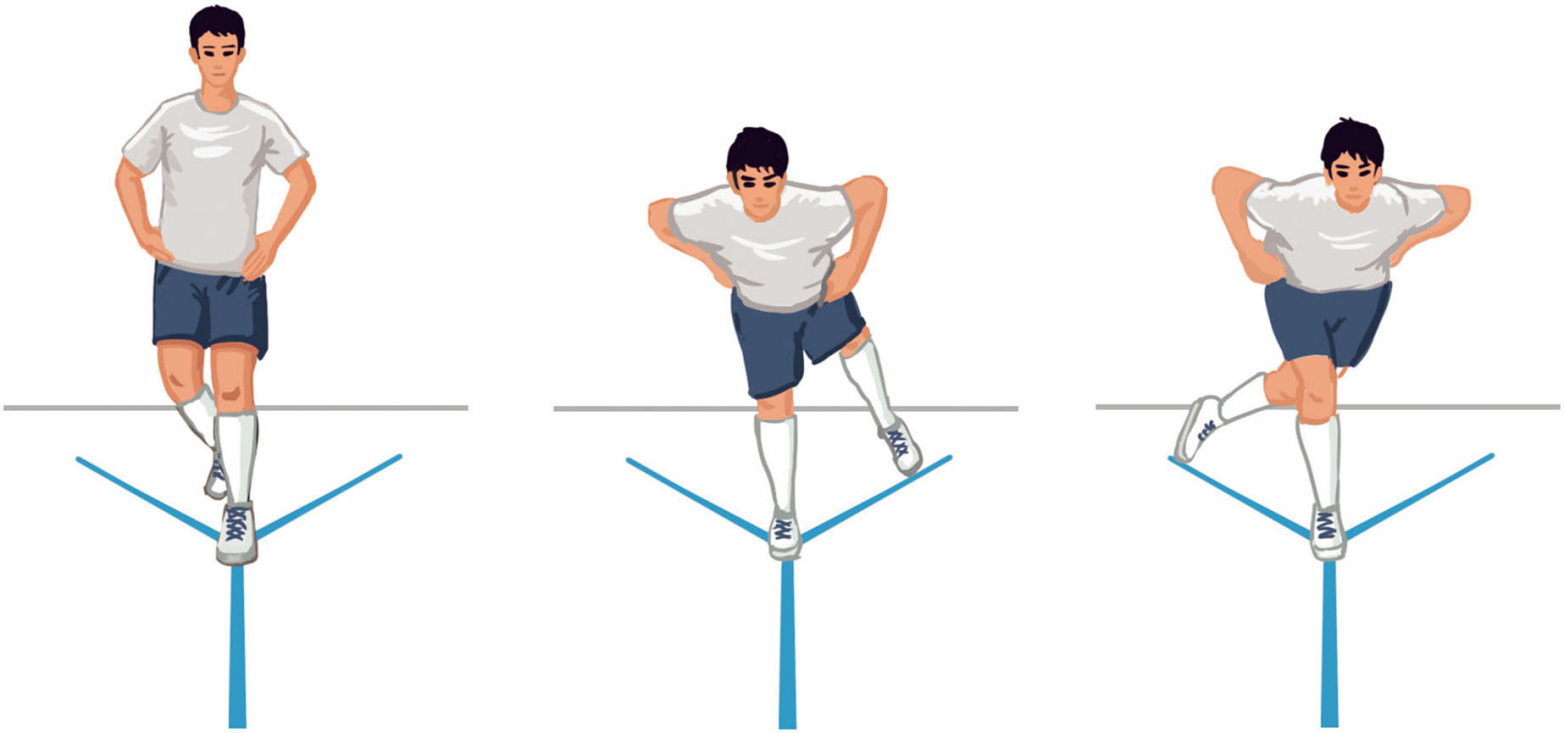
Y-balance test (YBT).

The test was used to assess the dynamic balance of players (Shaffer et al., 2013). While barefoot, participants balanced themselves with one foot on the center board of a commercially available YBT instrument (Move2Perform, Evansville, IN). To perform the test, they need to place their hands on the hips and reach as far as possible by pushing the board with the reaching limb into the anterior, posteromedial, and posterolateral directions and return to the original start position (see Figure 4 for the illustration). Reach distance was measured at the nearest edge of the reach indicator to the closest 0.5 cm. All participants performed three practice trials in each of three directions on each leg before three formals test trials. YBT reach distances were normalized to leg length (%), which was obtained by measuring the length from the right anterior superior iliac spine of the players to right medial malleolus in supine. The highest composite score was recorded for analysis using the following formula:

$$\begin{array}{l} \text{Composite Score}\\ =\frac{\left(\text{Anterior }+\text{ Posteromedial }+\text{ Posterolateral}\right)}{3\;\times \text{ Right Limb Length}}\times \;100 \end{array}$$

where anterior, posteromedial and posterolateral represent the distances reached in each direction (intraclass correlation coefficients: 0.92 [0.85, 0.96]).

### Reactive Strength Index Test

**Figure 5:**
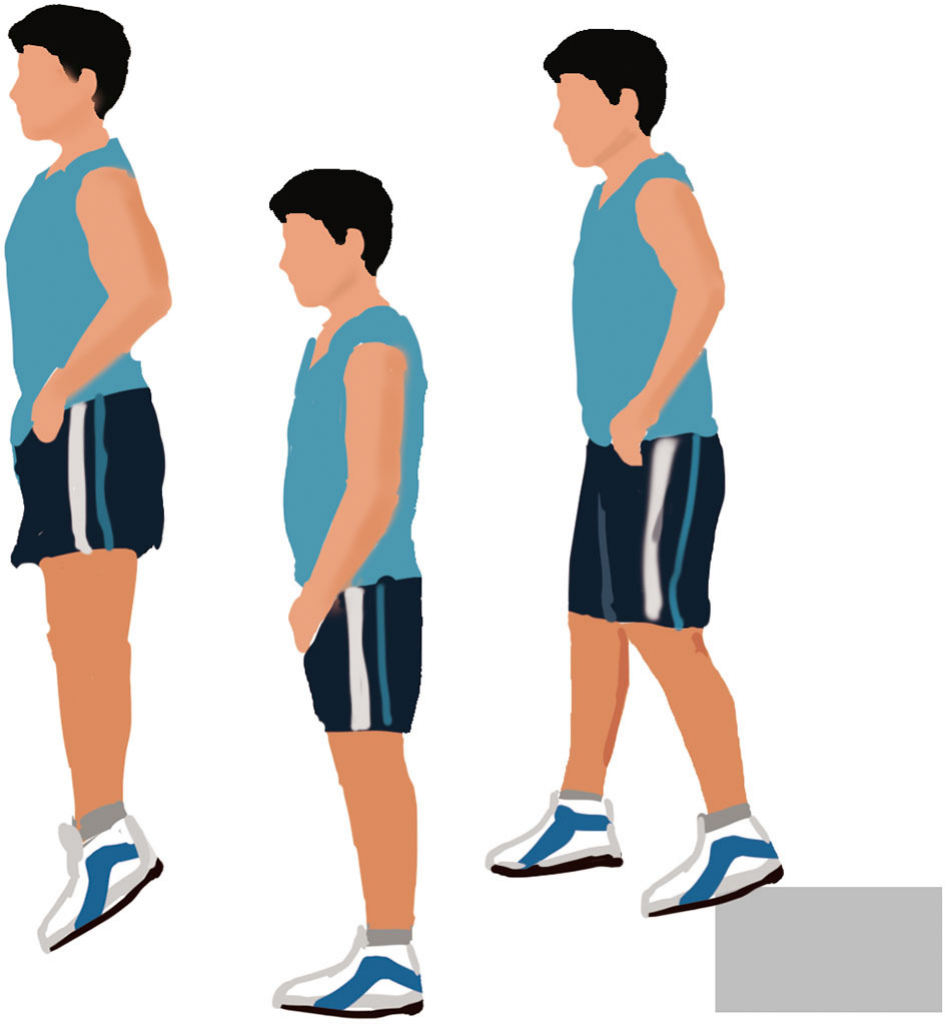
Reactive Strength Index (RSI) test.

The test is applied to evaluate how athletes perform during plyometrics by measuring the muscle-tendon stress and their reactive jump capacity so that their ability to quickly and effectively moving through the strength-shortening cycle is demonstrated (Ebben and Petushek, 2010). In practice, the RSI is strongly related to COD speed and acceleration speed and depth jump from the height of 30 cm could provide a valid result of RSI (Flanagan et al., 2008; Byrne et al., 2017). Therefore, in this study, the RSI was measured using depth jump from a 30 cm plyometric box (see Figure 5). During the test, participants were instructed to perform the depth jump with two hands on their hips and step forward off the box without stepping down or jumping up. After observing the demonstration, each player was then allowed to carry out two practice trials before formal testing. On falling, players would land on an in-ground force plate (Kistler 9281CA, Winterthur, Switzerland) that has a sampling frequency of 1,000 Hz. They were requested to jump as high and quickly as possible after landing, and the jump height was calculated from take-off velocity derived from their respective force-time data. Later, the RSI score was calculated using the highest jump height recorded from three trials using the below formula (intraclass correlation coefficients: 0.86 [0.75, 0.93]):

$$\begin{array}{l} \text{RSI}=\frac{\text{Jump height}}{\text{ground contact time}} \end{array}$$

### Statistical Analysis

Data are presented as means and SD. The normality of data distribution was confirmed using the Shapiro-Wilk test. Training-related effects were assessed by 2-way repeated-measure ANOVA (group × time) on SEMO Test, 5-0-5 COD test, YBT, and RSI, with the Greenhouse-Geisser adjustment was applied. Partial η^2^ was used as the effect size (ES) estimation for the time by group interaction effect with its strength being interpreted as the following: <0.06 as small, <0.14 as moderate, and ≥0.14 as large (Cohen, 1988), while the Cohen's *d* converted from partial η^2^ was used to represent the ES of main effect (Cohen, 1988). When a significant effect was found, Bonferonni *post-hoc* correction was performed to identify pairwise differences. The absolute value of each test result was used to calculate the ES for the within- and between-group comparisons, represented as Cohen's *d*. It was interpreted according to the following thresholds: <0.2 as trivial, 0.2–0.6 as small, 0.6–1.2 as moderate, 1.2–2.0 as large, and >2.0 as very large (Hopkins et al., 2009). The Statistical Package for Social Sciences (SPSS Inc., Chicago, IL, USA, version 22.0) was used for all analyses. The level of significance was set at *p* < 0.05 for all tests.

## Results

Table 3 presents the descriptive statistics of all COD tests, results of repeated ANOVA for pre-and post-training fitness testing, and corresponding ESs. No statistically significant differences between groups were found at baseline for all test measures.

**Table 3:** Descriptive statistics of agility test results for BP group and PL group before and after the 6-week training intervention.

	**BP group**			**PL group**			**Time**		Group^*^Time	
	**Pre**	**Post**	**ES**	**Pre**	**Post**	**ES**	***p***	**Cohen's *d***	***p***	**Partial η^2^**
SEMO agility test (s)	13.38 ± 0.45	12.78 ± 0.31	^*^1.55	13.37 ± 0.37	13.06 ± 0.38	^*^0.81	<0.001	1.49	0.159	0.14
5-0-5 COD test (s)	3.76 ± 0.25	3.44 ± 0.15	^*^1.55	3.72 ± 0.16	3.62 ± 0.17	^*^0.61	<0.001	3.03	0.001	0.58
YBT (dominant foot)	97.33 ± 5.32	104.94 ± 7.21	^*^1.20	96.16 ± 7.78	97.06 ± 7.75	0.12	0.002	1.16	0.008	0.38
YBT (non-dominant foot)	96.22 ± 5.58	104.78 ± 7.35	^*^1.31	96.08 ± 8.14	97.41 ± 6.53	0.18	<0.001	1.62	0.003	0.48
RSI	1.27 ± 0.14	1.50 ± 0.12	^*^1.76	1.22 ± 0.12	1.35 ± 0.15	^*^0.96	<0.001	2.52	0.045	0.26

* *Statistically significant difference between pre- and post-test, p < 0.05*.

*SEMO, modified southeast Missouri; 5-0-5 COD test = 5-0-5 change of direction test; YBT, Y-Balance test; RSI, reactive strength index*.

**Figure 6:**
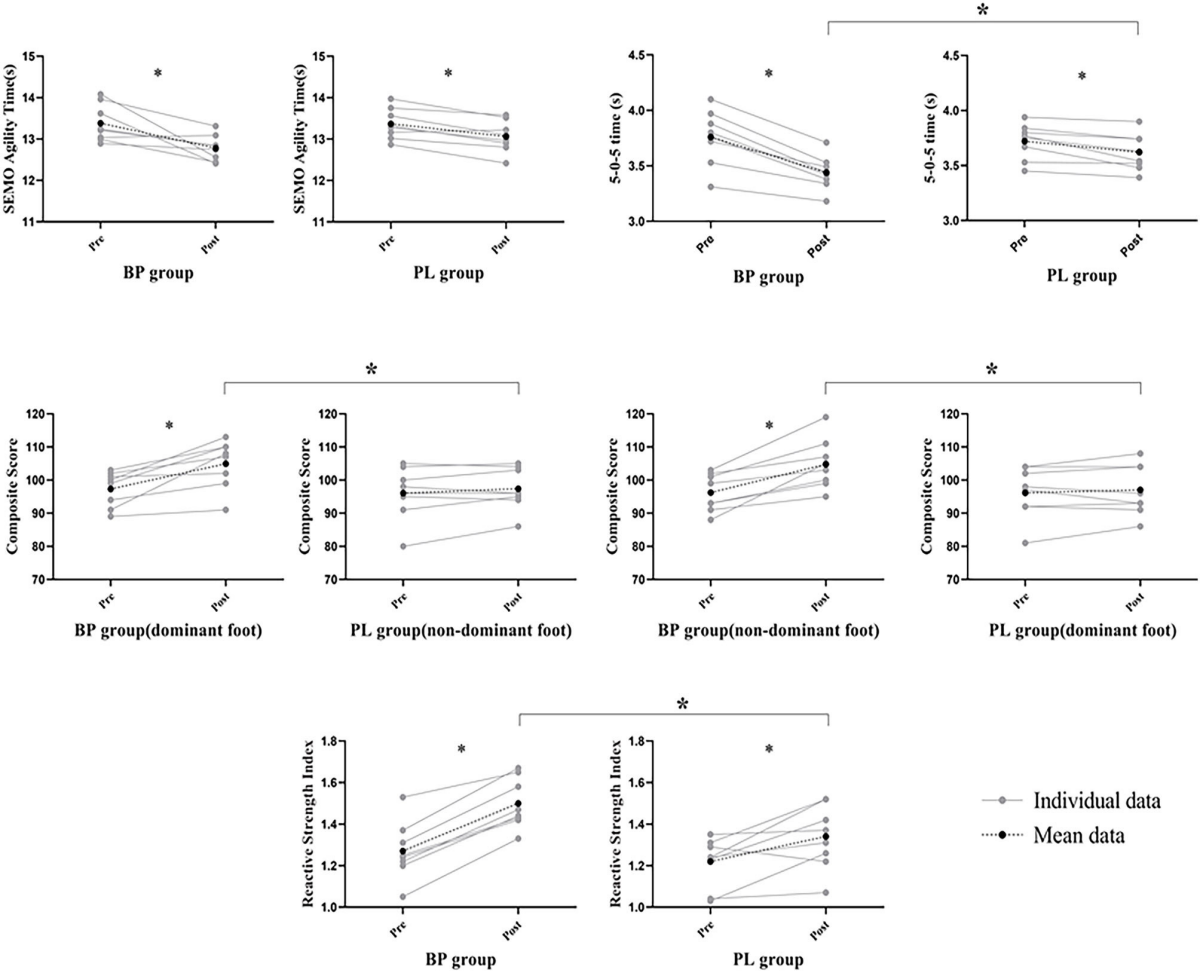
Individual and mean values for agility related tests during before and after Balance-Plyometric (BP) and Plyometrics (PL) training. Note: ^*^p < 0.05.

For both BP and PL groups, training resulted in statistically significant improvement in SEMO test, 5-0-5 COD test, and RSI (*p* < 0.001, partial η^2^ = 0.598, 0.835, and 0.783, respectively), but BP showed higher ESs than PL (1.55 vs. 0.81, 1.55 vs. 0.61, and 1.76 vs. 0.96). Significant group by time interaction effects were shown between groups for 5-0-5 COD test [*F*_(1, 14)_ = 19.273, *p* = 0.001, and partial η^2^ = 0.579], indicating a significantly greater improvement in the above test performance after the BP intervention when compared with the PL intervention. Moreover, a statistically significant increase in YBT was shown for the BP group on both dominant and non-dominant feet (ES = 1.20 and 1.31). Significant group by time interaction effects were shown between groups for YBT (dominant foot) [*F*_(1, 14)_ = 8.710, *p* = 0.008, and partial η^2^ = 0.384], YBT (non-dominant foot) [*F*_(1, 14)_ = 12.674, *p* = 0.003, and partial η^2^ = 0.475] and RSI [*F*_(1, 14)_ = 4.831, *p* = 0.045, and partial η^2^ = 0.257], indicating a significantly greater improvement in the above test performance after the BP intervention when compared with the PL intervention. Figure 6 depicts within- and between-group ESs for comparisons of test results.

## Discussion

The purpose of the study was to investigate the effectiveness of the 6-week combined balance and plyometric training program on the COD performance of elite badminton players. To the best of our knowledge, this is the first study comparing the effect between combined training and plyometric training. Based on the results, we found the combined protocol to be more effective in improving COD ability, dynamic balance, and RSI of players when compared with the latter. As COD ability, dynamic balance, and RSI are valid indicators of the movement performance of badminton players (Hughes and Bopf, 2005; Masu et al., 2014), the current findings could help enrich the badminton-specific strength and conditioning routines and improve the on-court performance.

Findings of the study imply that 6 weeks of BP training-induced better adaptations in COD, balance, and reactive strength performances. As the well-developed COD ability requires not only strong lower limb power to move quickly, but also a good balance to control body posture and to overcome the inertia caused by decelerations and brakings, adding an extra balance training indeed played an important role in boosting such performance (Sekulic et al., 2013). Former research documented a high correlation (r = 0.83) of COD ability with badminton performance during competitive matches (Tiwari et al., 2011), which are characterized by high-intensity rallies with short rest intervals (Alam et al., 2010). Although the directions and trajectories of shuttlecock are fixed during rallies, they are randomly determined by players at milliseconds of time before each stroke (Lees, 2003). Therefore, players are requested to execute rapid CODs, consecutive jumps, lunges, and multiple accelerations and decelerations (Manrique and Gonzalez-Badillo, 2003), and possessing decent COD is the key to applying the most efficient footwork into reaching the correct places and hitting the shuttlecock. As previously revealed that the most frequent moving patterns during badminton matches were forward-backward movements, lateral movements, and CODs (Phomsoupha and Laffaye, 2015), results of the SEMO Test and the 5-0-5 COD test would be a performance indicator of on-court COD of a player. Meanwhile, although sufficient investigations have already evidenced positive adaptations in the COD ability of athletes after plyometric training (Manouras et al., 2016; Asadi et al., 2017), the current study provided novel findings that a combined training could induce greater improvement, which is in consensus with findings of Bouteraa et al. (2020) when applying similar training methodology to female basketball players.

The results further revealed a significant increase in both Y-Balance and RSI performance for the BP group, while reporting only meaningful adaptation in RSI for the PL group. It could be inferred from this result that badminton-specific COD ability has been improved after plyometric training, but not as much as a combined training protocol, which is consistent with the previous research where significant adaptations were reported in female basketball players and ordinary adolescents after combined training (Chaouachi et al., 2014; Bouteraa et al., 2020). RSI is an indicator of how efficiently athletes perform the SSC. Although plyometric training has been shown to significantly improve the efficiency of SSC utilization (Jeffreys et al., 2019; Dallas et al., 2020), it is interesting to note that the BP group exhibited a greater increase in RSI. A potential reason could be that balance training enhanced the body control of players and finally reduced the time spent on the landing during jumps. It is also possible that balance training could be beneficial for the power improvement of the lower limbs. Kean et al. (2006) found a 10% increase in vertical jumping after 5-week balance training, which helped decrease the sway of the COG, allowing athletes to land more consistently with optimal vertical jump angles. Lower limb reactive strength and posture control are important factors affecting quick COD of athletes, body stability as well as effective prevention of injuries (Borghuis et al., 2008). In this regard, the combined training paradigm could be more preferable for the complex, high-speed, unilateral, repetitive dynamic badminton movements that request a high extent of postural control.

To understand the benefit of combined training to badminton performance from a physiological point of view, the coordinated work of the visual system, vestibular system, and proprioceptive system should be addressed. When a player is changing his directions quickly and the body is imbalanced, the aforementioned systems will work together to regain the balance and maintain body stiffness. During this process, the proprioceptors (muscle spindles and Golgi tendons) and other joint receptors will sense changes in body posture, and then the vestibular system will send feedback to the central nervous system to generate kinesthetic awareness. Subsequently, in order to control body balance, the central nervous system needs to transmit signals to effectors (muscles), which will ultimately respond appropriately by adjusting the COG of the body (Sekulic et al., 2013). Therefore, the ability to change direction and make fast acceleration/deceleration is attributed to the joint effort of proprioceptors and effectors. In essence, the plyometric training could only strengthen the function of the effector (muscle), but not the proprioceptive system, which would be improved *via* balance training. Therefore, the combined balance and plyometric training should be prioritized in boosting both the performance of the proprioceptive system and muscle function simultaneously.

The results of our study evidenced the promising effects of the combined training paradigm on badminton COD ability so that it seems feasible to apply balance training into the already existed plyometric training routines of players. Moreover, from a practical perspective, fitness coaches are suggested to vary the format and sequence of exercises, training volumes, and intensities of such training prescription in order to add more stimuli. For example, instead of separating plyometrics and balance training apart, coaches could adopt a circuit training protocol that involves two training modalities in each circuit, using balance training as an alternative for passive rest between different sets of exercises. Finally, coaches and sports scientists are suggested to investigate whether incorporating reaction training drills to combined training protocol could further improve badminton on-court COD performance.

Despite the new information about the effect of combined training on badminton COD ability, several limitations need to be acknowledged. First, due to the fact that most elite male badminton players usually have fixed match and training planning (traveling to compete and train) that are not possible to be alternated, the study could only include a comparatively small sample size. Second, it should be noted that an experiment period of 6 weeks was chosen in order to verify that a shorter intervention than the commonly-used 8 weeks to induce favorable adaptation, as the experiment schedule had to be acceptable for both the coaches and players. Researchers should be, therefore, cautious when interpreting and generalizing the current findings.

## Conclusions

In summary, the 6-week training intervention for elite male badminton players showed that combined training (BP) induced an overall better adaptation for the SEMO TEST, the 5-0-5 COD test, the YBT, and the RSI test than plyometric training (PL). Moreover, considering the completion of balance training is relatively time-efficient (20 min in the current study), it is suggested that adding such exercises would allow optimal training adaptation for badminton players.

## Data Availability Statement

The raw data supporting the conclusions of this article will be made available by the authors, without undue reservation.

## Ethics Statement

The studies involving human participants were reviewed and approved by Beijing Sport University Institutional Research Commission (Approval number: 2020008H). The patients/participants provided their written informed consent to participate in this study.

## Author Contributions

ZG, YH, and DB: conceptualization. ZG, YH, YC, and DB: methodology, validation, and writing—original draft preparation. ZG and YC: software and visualization. ZG, WG, and YC: formal analysis. ZG, ZZ, and BL: investigation. BL and DB: resources. ZG and YH: data curation. ZG, YH, and YC: writing—review and editing. DB and YC: supervision, project administration, and funding acquisition. All authors have read and agreed to the published version of the manuscript.

## Conflict of Interest

The authors declare that the research was conducted in the absence of any commercial or financial relationships that could be construed as a potential conflict of interest.
